# Handgrip strength an easy screening for cognitive decline in Primary Care old patients

**DOI:** 10.1093/geroni/igaf122.2440

**Published:** 2025-12-31

**Authors:** Constanca Paul, Susana Sousa, Laetitia Teixeira, Carla Tortora

**Affiliations:** University of Porto, Porto, Porto, Portugal; University of Porto, Porto, Porto, Portugal; ICBAS-UP, Porto, Porto, Portugal; University degli Studi “G. d’Annunzio” Chieti-Pescara, Chieti, Abruzzo, Italy

## Abstract

Hand grip strength (HGS) is considered a measure of overall muscular strength connected with mobility, functionality and frailty. In adition, HGS also appears associated with cognition, namely mild cognitive impairment (MCI) and Alzheimer’s disease. The early diagnosis of cognitive decline should be done in Primary Care(PC) allowing interventions to avoid or retard the progression to dementia. HGS is a quick and objective measure that seems to be be useful as a predictor of cognitive decline in Primary Care. The objective of this study is to better understand the association between HGS and usual screenings of Mild Cognitive Impairment MCI (Qmci) and dementia (MMSE). The participants of the study are 427 older adults, clients of PC. Together with cognitive tests and demographic variables, HGS was measured. The mean age of participants was 75.65 years (sd 6,76); 57.1% females with a mean of education’ years of 3.3 (sd 2.4). The HGS mean of men was 27.1(sd 8.8) and of women 14.5(sd 4.8). HGS predicts Qmci results (B = 24.69 p<.001) explaining 12.5% of variance and MMSE results (B = 18.37 p<.001), explaining 13.7% of variance. Data shows that lower HGS grip is associated with a higher risk of MCI and dementia. HGS is a simple measure allowing risk-stratification and should be used in regular secreening of older adults in PC and those with lower scores being refered to further cognitive assessment and a costumised physical activity intervention that will benefit the overall muscular strength and contribute to avoid or retard the onset of dementia.

